# Spatio‐temporal assessment of illicit drug use at large scale: evidence from 7 years of international wastewater monitoring

**DOI:** 10.1111/add.14767

**Published:** 2019-10-23

**Authors:** Iria González‐Mariño, Jose Antonio Baz‐Lomba, Nikiforos A. Alygizakis, Maria Jesús Andrés‐Costa, Richard Bade, Leon P. Barron, Frederic Been, Jean‐Daniel Berset, Lubertus Bijlsma, Igor Bodík, Asher Brenner, Andreas L. Brock, Daniel A. Burgard, Erika Castrignanò, Christophoros E. Christophoridis, Adrian Covaci, Pim de Voogt, Damien A. Devault, Mário J. Dias, Erik Emke, Despo Fatta‐Kassinos, Ganna Fedorova, Konstantinos Fytianos, Cobus Gerber, Roman Grabic, Stefan Grüner, Teemu Gunnar, Evroula Hapeshi, Ester Heath, Björn Helm, Félix Hernández, Aino Kankaanpaa, Sara Karolak, Barbara Kasprzyk‐Hordern, Ivona Krizman‐Matasic, Foon Yin Lai, Wojciech Lechowicz, Alvaro Lopes, Miren López de Alda, Ester López‐García, Arndís S. C. Löve, Nicola Mastroianni, Gillian L. McEneff, Rosa Montes, Kelly Munro, Thomas Nefau, Herbert Oberacher, Jake W. O'Brien, Kristin Olafsdottir, Yolanda Picó, Benedek G. Plósz, Fabio Polesel, Cristina Postigo, José Benito Quintana, Pedram Ramin, Malcolm J. Reid, Jack Rice, Rosario Rodil, Ivan Senta, Susana M. Simões, Maja M. Sremacki, Katarzyna Styszko, Senka Terzic, Nikolaos S. Thomaidis, Kevin V. Thomas, Ben J. Tscharke, Alexander L. N. van Nuijs, Viviane Yargeau, Ettore Zuccato, Sara Castiglioni, Christoph Ort

**Affiliations:** ^1^ Institute for Food Analysis and Research, Department of Analytical Chemistry Universidade de Santiago de Compostela Santiago de Compostela Spain; ^2^ Faculty of Chemical Sciences, Department of Analytical Chemistry, Nutrition and Bromatology University of Salamanca Salamanca Spain; ^3^ Norwegian Institute for Water Research (NIVA) Oslo Norway; ^4^ Department of Chemistry, Laboratory of Analytical Chemistry National and Kapodistrian University of Athens Athens Greece; ^5^ Food and Environmental Safety Research Group University of Valencia Moncada Spain; ^6^ School of Pharmacy and Medical Sciences University of South Australia Adelaide South Australia Australia; ^7^ King's Forensics School of Population Health and Environmental Sciences, King's College London London UK; ^8^ KWR Water Research Institute Nieuwegein the Netherlands; ^9^ University of Bern, Institute of Plant Sciences Bern Switzerland; ^10^ Research Institute for Pesticides and Water, University Jaume I Castellón Spain; ^11^ Department of Environmental Engineering, Faculty of Chemical and Food Technology Slovak University of Technology Bratislava Slovakia; ^12^ Unit of Environmental Engineering Ben‐Gurion University of the Negev Beer‐Sheva Israel; ^13^ Department of Environmental Engineering Technical University of Denmark Kongens Lyngby Denmark; ^14^ University of Puget Sound Tacoma WA USA; ^15^ Department of Chemistry University of Bath Bath UK; ^16^ Department of Analytical, Environmental and Forensic Sciences King's College London London UK; ^17^ Environmental Pollution Control Laboratory, Chemistry Department Aristotle University of Thessaloniki Thessaloniki Greece; ^18^ Department of Pharmaceutical Sciences Toxicological Center Antwerp Belgium; ^19^ IBED University of Amsterdam Amsterdam the Netherlands; ^20^ Université Paris‐Sud, CNRS, AgroParisTech, Université Paris‐Saclay Chatenay‐Malabry France; ^21^ National Institute of Legal Medicine and Forensic Sciences Lisbon Portugal; ^22^ NIREAS‐International Water Research Center, Department of Civil and Environmental Engineering University of Cyprus Nicosia Cyprus; ^23^ Faculty of Fisheries and Protection of Waters University of South Bohemia in Ceske Budejovice Zatisi Czech Republic; ^24^ Chair of Urban Water Management Technische Universität Dresden Dresden Germany; ^25^ Forensic Toxicology National Institute for Health and Welfare (THL) Helsinki Finland; ^26^ Department of Environmental Sciences Jožef Stefan Institute Ljubljana Slovenia; ^27^ Division for Marine and Environmental Research Rudjer Boskovic Institute Zagreb Croatia; ^28^ Department of Aquatic Sciences and Assessment Swedish University of Agricultural Sciences (SLU) Uppsala Sweden; ^29^ Institute of Forensic Research Krakow Poland; ^30^ Faculty of Pharmacy University of Lisbon Lisbon Portugal; ^31^ Water and Soil Quality Research Group, Department of Environmental Chemistry Institute of Environmental Assessment and Water Research (IDAEA‐CSIC) Barcelona Spain; ^32^ Department of Pharmacology and Toxicology University of Iceland Reykjavík Iceland; ^33^ Institute of Legal Medicine and Core Facility Metabolomics Medical University of Innsbruck Innsbruck Austria; ^34^ Queensland Alliance for Environmental Health Sciences (QAEHS) The University of Queensland Woolloongabba QLD Australia; ^35^ Department of Chemical Engineering University of Bath Bath UK; ^36^ Process and Systems Engineering Center (PROSYS), Department of Chemical and Biochemical Engineering Technical University of Denmark Kongens Lyngby Denmark; ^37^ Faculty of Technical Sciences, Department of Environmental Engineering and Occupational Safety University of Novi Sad Novi Sad Serbia; ^38^ Department of Coal Chemistry and Environmental Sciences AGH University of Science and Technology Krakow Poland; ^39^ Department of Chemical Engineering McGill University Montreal, Quebec Canada; ^40^ Istituto di Ricerche Farmacologiche Mario Negri IRCCS Milan Italy; ^41^ Eawag, Urban Water Management Swiss Federal Institute of Aquatic Science and Technology Dübendorf Switzerland

**Keywords:** Amphetamine, cocaine, ecstasy/MDMA, illicit drugs, methamphetamine, wastewater‐based epidemiology

## Abstract

**Background and aims:**

Wastewater‐based epidemiology is an additional indicator of drug use that is gaining reliability to complement the current established panel of indicators. The aims of this study were to: (i) assess spatial and temporal trends of population‐normalized mass loads of benzoylecgonine, amphetamine, methamphetamine and 3,4‐methylenedioxymethamphetamine (MDMA) in raw wastewater over 7 years (2011–17); (ii) address overall drug use by estimating the average number of combined doses consumed per day in each city; and (iii) compare these with existing prevalence and seizure data.

**Design:**

Analysis of daily raw wastewater composite samples collected over 1 week per year from 2011 to 2017.

**Setting and Participants:**

Catchment areas of 143 wastewater treatment plants in 120 cities in 37 countries.

**Measurements:**

Parent substances (amphetamine, methamphetamine and MDMA) and the metabolites of cocaine (benzoylecgonine) and of Δ^9^‐tetrahydrocannabinol (11‐nor‐9‐carboxy‐Δ^9^‐tetrahydrocannabinol) were measured in wastewater using liquid chromatography–tandem mass spectrometry. Daily mass loads (mg/day) were normalized to catchment population (mg/1000 people/day) and converted to the number of combined doses consumed per day. Spatial differences were assessed world‐wide, and temporal trends were discerned at European level by comparing 2011–13 drug loads versus 2014–17 loads.

**Findings:**

Benzoylecgonine was the stimulant metabolite detected at higher loads in southern and western Europe, and amphetamine, MDMA and methamphetamine in East and North–Central Europe. In other continents, methamphetamine showed the highest levels in the United States and Australia and benzoylecgonine in South America. During the reporting period, benzoylecgonine loads increased in general across Europe, amphetamine and methamphetamine levels fluctuated and MDMA underwent an intermittent upsurge.

**Conclusions:**

The analysis of wastewater to quantify drug loads provides near real‐time drug use estimates that globally correspond to prevalence and seizure data.

## Introduction

The global illicit drug market is estimated to be a hundred‐billion activity that facilitates corruption, affects the economic development of certain regions in the world 1, 2, and contributes to the global burden of disease 3. In economically developed regions, the disease burden from illicit psychoactive substance use is higher than in less developed regions and, compared to legal substances such as alcohol and tobacco, inflicts mortality earlier in life 4.

Determining the scale of the illicit drug market and its temporal dynamics is an important but challenging task for law and drug enforcement agencies to assess the efficacy of drug‐related policy and control/prevention measures. Historically, this has been established through a combination of seizures, surveys, drug treatment demands, drug‐related hospital admissions and arrest data. Wastewater‐based epidemiology (WBE) uses the analysis of illicit drug residues in wastewater to provide a quantitative measure of the mass loads of a drug released in a specific sewer catchment. Mass loads are normalized by the population size to provide the daily load released per 1000 people. Uncertainties associated with WBE measurements of drug loads, derived from in‐sewer phenomena, sewage sampling and analysis and population size estimations, are typically < 20% when studies are performed following the best practice protocol 5, 6 developed by the Sewage Analysis CORe Group Europe 7. Estimates of drug consumption are affected by additional sources of uncertainty, i.e. excretion factors, mass doses and drug purity (Supporting information, Appendix S1).

**Figure 1:**
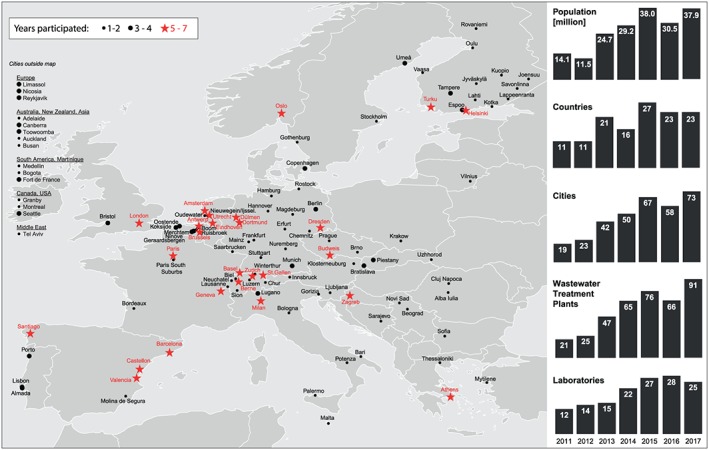
Participation in terms of (millions of) population covered and number of countries, cities, wastewater treatment plants and laboratories participating per year. Cities with a red star provided data for at least 5 years during 2011–17 [Colour figure can be viewed at http://wileyonlinelibrary.com]

In 2011, WBE was applied in the first international assessment of illicit drug use scenario in 19 European cities through the analysis of residues of five selected illicit drugs [cocaine, cannabis, amphetamine, methamphetamine and methylenedioxymethamphetamine (MDMA)] in raw wastewater 8. The monitoring was repeated every year to expand the spatial coverage and obtain consistent long‐term data 9. The number of cities increased from 19 (covering 14.1 million people) in 2011 to 73 (covering 37.9 million people) in 2017 (Fig. 1), and the monitoring was extended to Australia (AU), New Zealand (NZ), Colombia (CO), Martinique (MQ), Canada (CA), the United States (US), South Korea (KR) and Israel (IL). Thus, the aims of this study were to: (i) assess spatial and temporal trends in drug use by measuring benzoylecgonine, amphetamine, methamphetamine and MDMA mass loads in raw wastewater throughout 7 years; and (ii) address overall drug use by estimating the average number of combined doses consumed per day in each city. Results of 11‐nor‐9‐carboxy‐Δ^9^‐tetrahydrocannabinol (THC‐COOH, metabolite of Δ^9^‐tetrahydrocannabinol) are provided in Supporting information, Appendix S1 due to the challenges of its quantification in wastewater, which were assessed during the 7 years of study and, therefore, do not lead to readily comparable drug use figures 10.

## Methods

### Sampling

Every wastewater treatment plant (WWTP) provided aliquots of composite samples from the influent, representing raw wastewater over a 24‐hour period. Typically, samples were obtained for 7 consecutive days in March or April every year. A ‘normal’ week was targeted, avoiding special events such as public holidays or festivals. Population size, percentage of city population covered by each WWTP, sampling mode and dates are provided in Tables S1 and S2 of Supporting information, Appendix S1. The ISO 3166‐1 alpha‐2 code is used there and throughout the text to abbreviate country names. Figure 1 shows the level of participation per year. During the first 3 years (2011–13 8, 9) only European cities were monitored while, from 2014 onwards, cities in AU, NZ, CO, MQ, CA, US, KR and IL also participated in the sampling campaign (Fig. 1). During the reporting period, wastewater from more than 60 million people, connected to 143 individual WWTPs in 120 cities in 37 countries, was analysed at least once over 1 week. Twenty‐six cities from 14 European countries (29 WWTPs with approximately 19.3 million people connected) provided data for 5 or more years, building a core data set essential to assess temporal changes. A questionnaire was sent to WWTP managers each year before the start of the sampling campaign to gather information on WWTP catchment areas, appropriateness of sampling and details of the monitoring period 6.

### Analytical methodology

The analytical procedures applied for the determination of illicit drugs and their metabolites in wastewater have changed little since the first study in 2011 8, except for certain modifications and improvements derived from a better understanding of the fate of biomarkers in sewers 11, particularly in the case of THC‐COOH 10. Participants employed validated analytical methodologies, which generally consisted of: (i) spiking samples with stable isotope‐labelled internal standards (SILIS) for each analyte, in order to correct for matrix interferences and/or losses during sample treatment; (ii) filtration or centrifugation of samples to remove solid particles; (iii) off‐line solid‐phase extraction (SPE) for pre‐concentration and clean‐up; and (iv) analysis by liquid chromatography coupled to tandem mass spectrometry (LC–MS/MS). More details on analytical methodologies are available in Hernández *et al*. 12.

### Load, dose calculations and comparison with established drug use indicators

**Figure 2:**
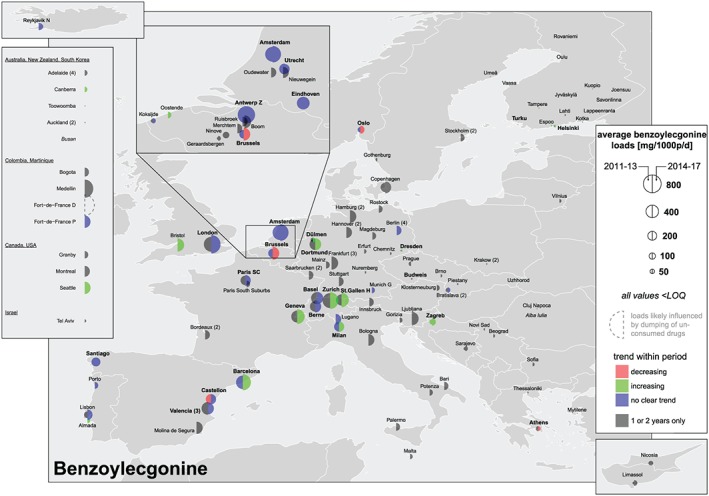
Mean population‐normalized benzoylecgonine loads (mg/1000 people/day) 2011–13 versus 2014–17 [Colour figure can be viewed at http://wileyonlinelibrary.com]

**Figure 3:**
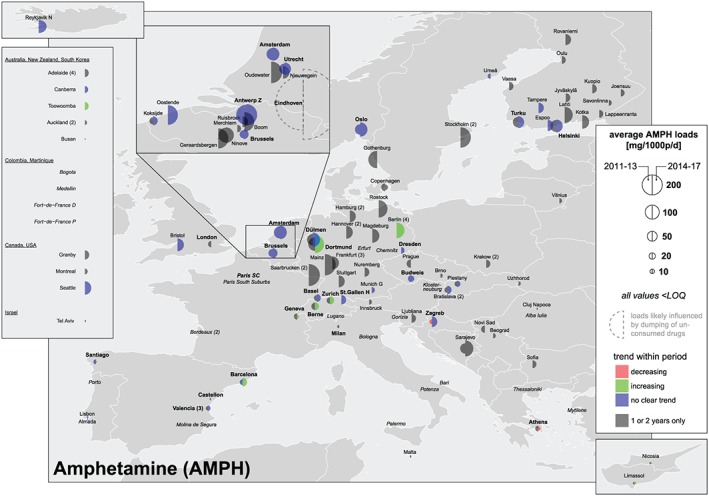
Mean population‐normalized amphetamine loads (mg/1000 people/day) 2011–13 versus 2014–17 [Colour figure can be viewed at http://wileyonlinelibrary.com]

**Figure 4:**
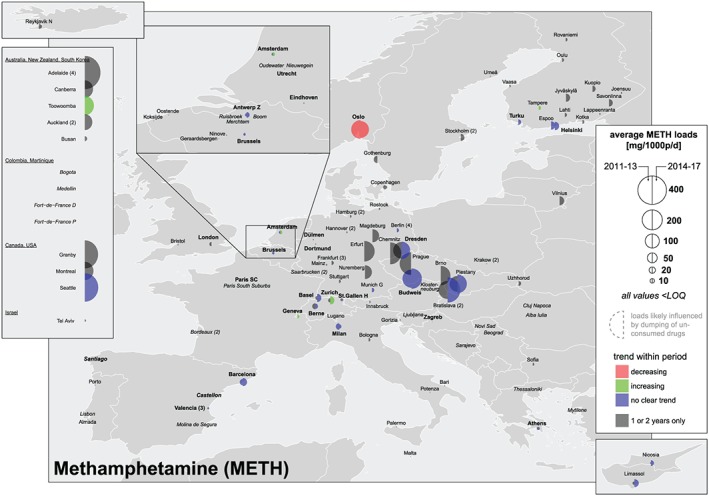
Mean population‐normalized methamphetamine loads (mg/1000 people/day) 2011–13 versus 2014–17 [Colour figure can be viewed at http://wileyonlinelibrary.com]

**Figure 5:**
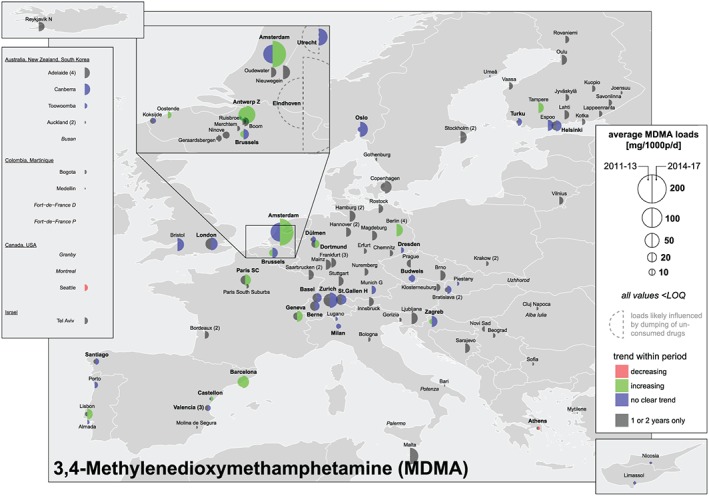
Mean population‐normalized 3,4‐methylenedioxymethamphetamine (MDMA) loads (mg/1000 people/day) 2011–13 versus 2014–17 [Colour figure can be viewed at http://wileyonlinelibrary.com]

Parent substances (amphetamine, methamphetamine and MDMA) and two urinary metabolites (benzoylecgonine for cocaine and THC‐COOH for cannabis) were measured in influent wastewater. Concentrations (ng/l) were multiplied by wastewater daily flow rates (l/day) and divided by the population served by each WWTP to gain population‐normalized loads (mg/1000 people/day). Means, standard deviations, maximum and minimum values for every substance, city and year are available through an open on‐line repository 13. These figures are not provided for regions (i.e. North or South Europe) due to the coexistence of cities with very low and very high population‐normalized loads in the same region. Alternatively, two types of overall means were calculated: (i) all cities in a specific year (dashed lines on the right side of figures in Supporting information, Appendix S2); and (ii) cities that provided data for 5 or more years (dotted lines). Locations were excluded from the overall mean calculation if: (i) all concentrations within a week were < LOQ (i.e. below the limit of quantification of the method); and (ii) an abnormally high or low value was reported for at least 1 year [e.g. Eindhoven (NL) for amphetamine and MDMA; see sections on ‘Amphetamine’ and ‘MDMA’]). Cities reporting at least one concentration > LOQ within 1 week were considered by replacing values < LOQ by 0.5 × LOQ. Maps and graphs summarizing all results were created using R 14. Figures 2, 3, 4, 5 allow: (i) gaining a quick spatial overview, (ii) comparing new (2014–17) and previously published data (2011–13 8, 9) by the size of the semicircles; and (iii) assessing temporal trends within each period by the colour of the semicircles. An increase or decrease was assigned if the slope from a linear regression was significantly different from zero (*P* < 0.2). No trend was assigned if only two observations were available within a period or if *P* was > 0.2.

Average excretion rate coefficients for each metabolite/residue 15, 16 and average doses of the parent drug (Table S3 of Supporting information, Appendix S1 17, 18) were applied to population‐normalized mass loads to gain an estimated number of pure doses consumed per day. Doses of cocaine, amphetamine, methamphetamine and MDMA were then summed to acquire the number of combined doses consumed per day. Amphetamine use was estimated from the entire loads of this compound, despite other sources that may contribute to its presence in wastewater: amphetamine disposal, licit‐prescribed use of amphetamine or methamphetamine metabolism. Accordingly, methamphetamine consumption was derived from methamphetamine loads solely. Cannabis (estimated from THC‐COOH) was excluded from combined doses calculations due to the higher uncertainty in its WBE‐derived use estimations 10, 19. Results for THC‐COOH are provided in Supporting information, Appendices S1 and S2.

European WBE results were compared to established epidemiological indicators of drug use, i.e. seizure statistics, purity and price data and prevalence estimates derived from population surveys and indirect statistical methods 1, 2. As the information provided by these indicators and WBE is not directly comparable, a qualitative analysis showed the points of agreement/disagreement and the potential complementarity of both methodologies. Results from cities outside Europe were excluded from these analyses due to the limited number of sites and years monitored, and were insufficient to extrapolate spatial and temporal trends.

### Uncertainties related to WBE

WBE data are subject to different uncertainties (Table S4 of Supporting information, Appendix S1). While participating laboratories’ performance of chemical analysis was systematically checked through yearly interlaboratory studies 19, 20, not all other aspects could be quantified accurately in such a large‐scale study with reasonable efforts. However, with spatial differences of WBE results spanning more than two orders of magnitude among locations for all substances, uncertainties seem to play a subordinate role.

Random uncertainties mainly affect the assessment of temporal changes within one location. In view of monitoring 1 week per year only, apparent short‐term trends should be interpreted with caution.

Systematic uncertainties, e.g. inaccurate population size or neglecting in‐sewer transformation, can lead to systematic under‐ or overestimation. This affects assessing spatial differences of drug residues in sewers and calculation of consumption estimates. In‐sewer processes (exfiltration and transformation) would lead to an underestimation of drug loads entering the sewer system. Recent laborious laboratory and full‐scale studies indicate that this underestimation is smaller than 10% for benzoylecgonine, methamphetamine and MDMA for typical hydraulic residence times (< 12 hours) under most conditions 15, 21. Only amphetamine is susceptible to higher transformations, which may lead to a site‐specific underestimation of consumption, as a global correction factor cannot be applied. Unconsumed cocaine dumped into sewers would lead to elevated benzoylecgonine loads. This can be discovered with abnormally high cocaine to benzoylecgonine ratios, as not all cocaine will transform to benzoylecgonine.

## Results

### Cocaine

Benzoylecgonine, a biomarker of cocaine consumption, was one of the substances measured at highest levels in European wastewaters during the 7 years. Wide spatial differences observed during the early monitoring campaigns in 2011–13 8, 9 were confirmed in 2014–17 (Fig. 2). Population‐normalized loads were generally higher in southern and western cities compared to eastern and northern locations. The highest weekly mean values (600–900 mg benzoylecgonine/1000 people/day) were found in London (UK), Bristol (UK), Amsterdam (NL), Zurich (CH), Geneva (CH), St Gallen (CH) and Antwerp (BE) (Supporting information, Appendix S2). In most of the countries where several locations were studied (BE, NL, DE, CH, ES), population‐normalized loads were higher in large cities compared to smaller towns (Supporting information, Appendix S2). This pattern had already been reported by Ort *et al*. 9 and had been observed in national studies in BE 22, FR 23, IT 24, CH 25 and FI 26.

In terms of temporal trends, population‐normalized mean loads were higher (depicted with a larger semicircle) in 2014–17 compared to 2011–13 in Barcelona (ES), Lisbon (PT), Geneva (CH), Zurich (CH), St Gallen (CH), Zagreb (HR), Bratislava (SK), Brussels (BE), Amsterdam (NL), London (GB), Copenhagen (DK) and Oslo (NO). In nine cities (Barcelona, Geneva, Zurich, St Gallen, Bristol, Milan, Dortmund, Dulmen and Zagreb) a significant increasing trend was also observed during the last 4 years (green semicircle). When considering only the cities that provided data for at least 5 years, overall mean benzoylecgonine loads increased from 281–331 mg/1000 people/day in 2011–13 to 329–373 mg/1000 people/day in 2014–17 (dotted lines in Supporting information, Appendix S2).

Population‐normalized mass loads of benzoylecgonine were relatively high in South American locations compared to other regions outside Europe (Fig. 2). MQ is located close to the cocaine trade routes, which was reflected in the levels of benzoylecgonine measured in wastewater. The three North American cities showed a higher prevalence of cocaine use than the Australasian cities. In both regions, an increasing trend was observed in the participating locations with long‐term data.

### Amphetamine

In Europe, the highest population‐normalized mass loads of amphetamine were found in cities from BE and NL, in some cases exceeding by far the mean loads found in the rest of the continent (Fig. 3 and Supporting information, Appendix S2). The frequent high values measured in Eindhoven were attributed to direct discharges of drug manufacturing wastes 27, 28 and, consequently, excluded from the calculation of overall means (see section: ‘Load, dose calculations and comparison with established drug use indicators’). Loads reported in the Swedish cities and in Reykjavík (IS), despite being monitored for only 1–3 years, suggest a high use of amphetamine in northern European countries, matching the trend previously detected in some Finnish cities and in Oslo (NO) 29. DE also exhibited high loads of amphetamine, although with a great variation among cities. Comparatively, loads measured in southern European cities were much lower.

A significant increasing trend was observed within 2014–17 in Barcelona (ES), Geneva (CH), Berne (CH), Zurich (CH), Dortmund (DE) and Berlin (DE), and no decreasing trends were observed. However, overall population‐normalized mean loads from the cities that provided data for 5 or more years, including all these locations except Berlin, showed no apparent major change during 2011–17 (ca. 40 mg/1000 people/day, Supporting information, Appendix S2).

Outside Europe, amphetamine loads were typically low, and may be largely attributable to methamphetamine metabolism 30 and to the use of prescribed amphetamine 31.

### Methamphetamine

Although the average use of methamphetamine in Europe is low when compared to other stimulants, some localized hotspots were identified, mostly in eastern countries. Bratislava (SK), Piestany (SK), Prague (CZ), Budweis (CZ), Brno (CZ), Dresden (DE), Chemnitz (DE), Erfurt (DE) and Oslo (NO) showed the highest population‐normalized loads in wastewater, with weekly mean values exceeding 150 mg/1000 people/day (Supporting information, Appendix S2). Some cities in FI and CH reported year‐to‐year increases in the loads measured over 2014–17, although this increase was only statistically significant in Tampere (FI), Zurich (CH) and Geneva (CH). Interestingly, the opposite trend was shown in Oslo (NO), a city which had previously ranked very high regarding methamphetamine use (Fig. 4). Considering the overall means from locations providing data for 5 or more years, a decrease of more than 50% was observed from 2011 (39 mg/1000 people/day) to 2013 (18 mg/1000 people/day), followed by a steady increase up to 31 mg/1000 people/day in 2017 (Supporting information, Appendix S2).

Unlike the European overview, methamphetamine dominated the drug landscape in the cities monitored in North America (US and CA) and Australasia (AU, NZ and KR). Population‐normalized mass loads exceeded those in eastern Europe, where methamphetamine consumption is considered to be high (Fig. 4). In Busan (KR) methamphetamine loads were the highest among the drugs included in the study, although they were low compared to the values reported in North American, Australian and New Zealand cities. Tel Aviv (IL), Fort de France (MQ) and Colombian cities showed little evidence of methamphetamine consumption.

### MDMA

The highest population‐normalized mass loads of MDMA during the 7 years were reported in the Dutch cities of Eindhoven, Utrecht and Amsterdam (Fig. 5). Eindhoven and Utrecht were excluded from overall mean calculations and trend analyses due to the major impact of direct disposal events, which could originate from discharges under the pressure of police raids or fly‐tipping waste from illicit drugs synthesis 27. High loads were also measured in cities in BE, GB and CH, whereas eastern and southern European locations showed lower values. As in the case of cocaine, MDMA population‐normalized mass loads were usually higher in large cities, a trend observed in BE, CH and DE, but also in historically low‐MDMA‐usage countries such as ES, FR and PT (Fig. 5).

In terms of temporal variations, there was a higher number of cities where MDMA was quantified in 2016 and 2017 compared to earlier years. There was also an increasing trend in the loads measured, with many of the cities that were monitored for at least 5 years reporting an increase from 2011–13 to 2014–17; i.e. Helsinki (FI), Oslo (NO), Amsterdam (NL), Brussels (BE), Dortmund (DE), Zagreb (HR), Zurich (CH), Geneva (CH) and Barcelona (ES) (Fig. 5). However, this upsurge was non‐linear, and there were other large cities where MDMA loads decreased from 2011–13 to 2014–17, e.g. Milan (IT). Considering all the cities that provided data for 5 or more years, overall mean loads increased intermittently during 2011–17, reaching a maximum of 33 mg/1000 people/day in 2017 (Supporting information, Appendix S2).

Outside Europe, population‐normalized mass loads of MDMA were generally low (Fig. 5). Tel Aviv (IL) was the only city reporting relatively high MDMA levels compared to the other drugs. However, even there, MDMA use was low when compared to European sites.

### Consumption estimates: 2011–17 mean of sum of doses

**Figure 6:**
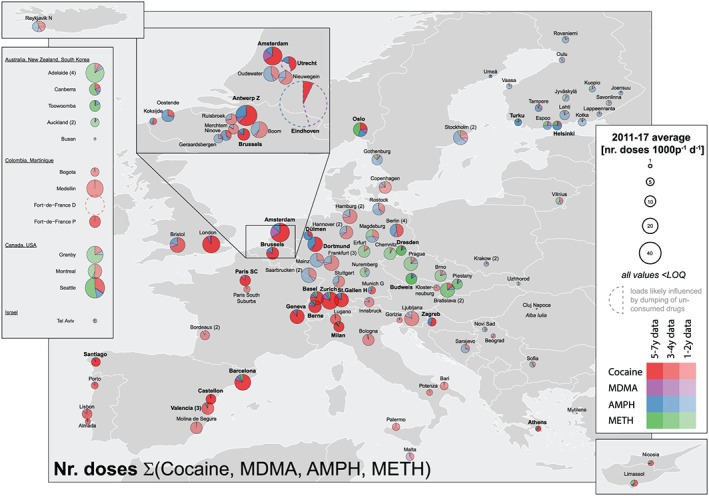
2011–17 total average number of doses/1000 people/day

Community drug use was assessed by calculating the number of combined doses of cocaine, amphetamine, methamphetamine and MDMA in each city. The highest numbers were found in Antwerp (BE), Amsterdam (NL), Zurich (CH), London (GB) and Barcelona (ES), with 43, 33, 28, 28 and 25 combined doses/1000 people/day, respectively (Fig. 6). Conversely, Athens (GR), Almada (PT), Joensuu (FI), Krakow (PL) and Umeå (SE), with three, three, two, two and one combined doses/1000 people/day, respectively, were some of the cities with the lowest rates. The average community drug use for all the investigated European cities was 13 doses/1000 people/day.

Considering the eight cities with available data during the 7 years, a U‐shaped curve was observed with 20 and 21 combined doses/1000 people/day in 2011 and 2017, respectively, and a minimum of 15 doses/1000 people/day between 2013 and 2015. Average combined doses for southwestern European cities were dominated by cocaine, while methamphetamine was the predominant drug measured in eastern sites. The consumption in the Nordic countries and in some German cities was mainly of amphetamine. Proportions of MDMA were relatively low for most cities excepting some locations in NL and FI.

Outside Europe, Medellín (CO), Adelaide (AU) and Seattle (US) had relatively high drug consumption (Fig. 6). However, Adelaide and Seattle combined doses were mainly indicative of methamphetamine use 32, 33, while Medellín was due to cocaine consumption, similarly to the situation in western Europe.

## Discussion

### Spatial differences

Among the substances investigated, the 2018 European Drug Report 34 identifies cocaine as the most prevalent and most frequently seized illicit stimulant in southern and western Europe. Conversely, amphetamines and MDMA are reported as the most frequently consumed stimulants in northern and eastern countries, where their seizures are also predominant 34. These spatial trends correspond to WBE results (Figs 2, 3, 4, 5, 6 and Supporting information, Appendix S2): (i) the highest population‐normalized mass loads of benzoylecgonine were measured in cities in southern and western countries (GB, NL, BE, CH, ES); (ii) the highest loads of amphetamine were observed in locations from the Nordic countries, DE and, especially, BE and NL; and (iii) the highest levels of MDMA were found in BE and NL. The 2016 Drug Markets Report 35 reports a market expansion of methamphetamine from the East to North and Central Europe, and a similar trend was observed in this study. The highest methamphetamine loads were found in eastern locations (CZ, SK and East German cities such as Chemnitz and Dresden), but high levels were also reported in Oslo (NO), and some cities in FI, CH and DE underwent a load increase during 2011–16. The location of DE in Central Europe confers it a special character in terms of drug usage, and trends in this country are better defined by regional geography than by national boundaries. Amphetamine and cocaine loads dominated the North and West of the country, in line with neighbouring cities in BE, NL, DK and SE; methamphetamine was found at higher levels in the East, reflecting a trend with neighbouring cities in CZ.

Amphetamine levels in wastewater need to be interpreted with caution in regions with high consumption of methamphetamine, such as North America, Australasia and eastern Europe. Following the administration of methamphetamine, approximately 4–7% is excreted as amphetamine 36, a percentage that may lead to non‐negligible amounts of this substance in wastewater 30. Similarly, if the prescription of amphetamine is high, levels derived from prescribed use need to be subtracted from those originated from illicit consumption, a correction not applied here. For instance, the relatively high levels of amphetamine found in this study in Seattle are probably related to its extended use to treat attention deficit/hyperactivity disorders in the United States 31, and not to its illicit consumption. Thus, further studies should separate the different sources contributing to the presence of a certain drug residue in wastewater. Amphetamine, methamphetamine and MDMA can also be directly disposed into sewer systems, and the amount of drug disposed needs to be differentiated from the amount excreted (i.e. consumed). This is currently possible through the application of chiral chromatography and ‘fingerprint’ analyses. Based on the enantiomerism and stereoselective metabolism of amphetamine derivatives, these tools have been used to identify dumping phenomena 27, 28, 37; characterize methamphetamine trafficking routes 38; differentiate between licit and illicit use of amphetamine 39; and distinguish amphetamine residues in wastewater derived from its consumption and from methamphetamine metabolism 37, 40.

### Temporal trends

The monitoring of 26 European cities for 5 or more years allowed discerning temporal trends in drug use. In some of these cities, population‐normalized mass loads of benzoylecgonine were not only comparatively higher in 2014–17 versus 2011–13, but also increased significantly within the last period (2014–17). This can indicate either a higher prevalence of cocaine consumption, a similar number of users consuming a higher amount of drug or an increase in the drug purity. Prevalence data suggest an overall stable use of cocaine throughout Europe and seizures have either remained unchanged or increased slightly in the last years, but cocaine purity rose on average ~32–54% from 2009 to 2016 34, 41.

For amphetamine and methamphetamine, an individual comparison of wastewater loads with other drug use indicators is hard to obtain, as both substances are not distinguished in many of the established indicator data sets. This highlights the relevance of WBE, which can complement classic epidemiological indicators with information on specific substances to jointly draw a more comprehensive picture of the drug use scenario. Thus, the fluctuations observed in amphetamine and methamphetamine loads in northern countries since 2011 26, 29 can reflect a changing market, with users taking one substance or the other depending on availability. Users are often unaware of which of the two drugs they are taking and, while methamphetamine use decreased in Oslo (NO) from 2011 to 2017, the combined use of amphetamine‐like stimulants remained stable.

Both established epidemiological indicators and wastewater data point to a recent revival of MDMA in Europe 34. The number of seizures has increased from approximately 13 000 in 2012 to 24 000 in 2016 (85% increase), while prevalence data suggest a stable or increased use of MDMA in the last years 34, 41. Matching this trend, the overall mean loads for the 26 cities providing data for 5 or more years increased intermittently since 2011, and the number of cities where MDMA was positively quantified in wastewater underwent an upsurge in 2016–17 compared to previous years. However, a factor that could contribute to these higher loads is the known increased content of MDMA in ecstasy tablets since 2010–11 42, 43.

### Combined doses

We acknowledge that back‐calculation of doses entails an additional degree of uncertainty in terms of differing and variable purity, different administration routes and different amounts of drug used over time and among different locations; however, we calculated the total number of combined doses because it provides important insights into the total scale and profile of drug use (Fig. 6). Cocaine prevalence dominates the southwest of Europe until the border with BE and CH, where other drug use patterns start to emerge, i.e. amphetamine towards the North and methamphetamine towards the East and Central Europe, the main historical production hotspots. As previously observed, these results reflect the profiles of use reported by other epidemiological indicators 34. Therefore, these complementary data can be used in the future to spatially define the impact area of each type of drug.

## Conclusions

This is the largest WBE study ever performed in terms of cities (120) and countries (37) involved and of the monitoring duration (2011–17). The extensive data set obtained for cocaine, amphetamine, methamphetamine and MDMA showed a comprehensive picture of spatial and temporal trends of use. Despite the limitation of monitoring few cities per country and comparing results with national statistics, the broader WBE picture corresponds to the epidemiological indicators considered, e.g. prevalence data and seizures statistics, demonstrating the capability of WBE to be used as an additional and complementary indicator of drug use.

WBE provides updated and objective estimates of drug use and allows identifying and highlighting new trends and specific profiles of use much earlier than other epidemiological indicators. WBE can serve as an extremely flexible tool for application at different spatial and temporal scales and can indicate mitigation measures nearly in real time. Thus, merging WBE results with information from other epidemiological indicators can improve our understanding of the drug use scenario.

## Declaration of interests

None.

## Supporting information


**Appendix S1** Information on WWTPs and population covered (Table S1); sampling modes and dates (Table S2); data used for the back‐calculation of combined doses (Table S3); uncertainty assessment (Table S4) and results for THC‐COOH.

**Appendix S2** Population‐normalized loads for benzoylecgonine, amphetamine, methamphetamine, MDMA and THC‐COOH and population‐normalized number of combined doses. In addition, an open online repository available at https://doi.org/10.25678/000172 compiles load results.
