# Supplementary material for: Spatio‐temporal assessment of illicit drug use at large scale: evidence from 7 years of international wastewater monitoring
Source: Addiction. 2019 Oct 23;115(1):109–20. doi: 10.1111/add.14767 (PMC6973045; doi:10.1111/add.14767)
Supplement: Supplementary file 1 — Appendix S1 Information on WWTPs and population covered (Table S1); sampling modes and dates (Table S2); data used for the back‐calculation of combined doses (Table S3); uncertainty assessment (Table S4) and results for THC‐COOH. [file ADD-115-109-s001.pdf]

Appendix 1

Spatio-temporal assessment of illicit drug use at large scale: evidence from seven years of international wastewater monitoring

Authors:

Iria González-Mariño[1,2,*], Jose Antonio Baz-Lomba[3]
Nikiforos A. Alygizakis[4], Maria Jesús Andrés-Costa[5], Richard Bade[6], Leon P. Barron[7], Frederic Been[8], Jean-Daniel Berset[9], Lubertus Bijlsma[10], Igor Bodík[11], Asher Brenner[12], Andreas L. Brock[13], Daniel A. Burgard[14], Erika Castrignanò[15,16], Christophoros E. Christophoridis[17], Adrian Covaci[18], Pim de Voogt[19], Damien A. Devault[20], Mário J. Dias[21], Erik Emke[8], Despo Fatta-Kassinos[22], Ganna Fedorova[23], Konstantinos Fytianos[17], Cobus Gerber[6], Roman Grabic[23], Stefan Grüner[24], Teemu Gunnar[25], Evroula Hapeshi[22], Ester Heath[26], Björn Helm[24], Félix Hernández[10], Aino Kankaanpaa[25], Sara Karolak[20], Barbara Kasprzyk-Hordern[15], Ivona Krizman-Matasic[27], Foon Yin Lai[28], Wojciech Lechowicz[29], Alvaro Lopes[30], Miren López de Alda[31], Ester López-García[31], Arndís S.C. Löve[32], Nicola Mastroianni[31], Gillian L. McEneff[7], Rosa Montes[1], Kelly Munro[7], Thomas Nefau[20], Herbert Oberacher[33], Jake W. O'Brien[34], Kristin Olafsdottir[32], Yolanda Picó[5], Benedek G. Plósz [13,35], Fabio Polesel[13], Cristina Postigo[31], José Benito Quintana[1], Pedram Ramin[13,36], Malcolm J. Reid[3], Jack Rice[15], Rosario Rodil[1], Ivan Senta[27], Susana M. Simões[21], Maja M. Sremacki[37], Katarzyna Styszko[38], Senka Terzic[27], Nikolaos S. Thomaidis[4], Kevin V. Thomas[3,34], Ben J. Tscharke[34], Alexander L.N. van Nuijs[18], Viviane Yargeau[39], Ettore Zuccato[40], Sara Castiglioni[40,*], Christoph Ort[41].

*Corresponding authors:
Iria González-Mariño: Tel: +34 881 816 035; e-mail: iria.gonzalez@usc.es, iriagonzalez@usal.es
Sara Castiglioni: Tel: +39 02 39014776; e-mail: sara.castiglioni@marionegri.it

1. Institute for Food Analysis and Research, Department of Analytical Chemistry, Universidade de Santiago de Compostela, 15782 Santiago de Compostela, Spain
2. Faculty of Chemical Sciences, Department of Analytical Chemistry, Nutrition and Bromatology, University of Salamanca, 37008 Salamanca, Spain
3. Norwegian Institute for Water Research (NIVA), Gaustadalléen 21, NO-0349 Oslo, Norway
4. National and Kapodistrian University of Athens, Department of Chemistry, Laboratory of Analytical Chemistry, 15771 Athens, Greece
5. Food and Environmental Safety Research Group (SAMA-UV) - CIDE (CSIC-UV-GV), University of Valencia, Moncada-Naquera Road Km 4,3, 46113 Moncada, Spain
6. School of Pharmacy and Medical Sciences, University of South Australia, GPO Box 2471, Adelaide, South Australia 5001, Australia
7. King’s Forensics, School of Population Health & Environmental Sciences, King's College London, London SE1 9NH, United Kingdom
8. KWR Watercycle Research Institute, P.O. Box 1072, 3430 BB, Nieuwegein, The Netherlands
9. University of Bern, Institute of Plant Sciences, Altenbergrain 21, 3013 Bern, Switzerland
10. Research Institute for Pesticides and Water, University Jaume I, Avda. Sos Baynat s/n, E-12071 Castellón, Spain
11. Department of Environmental Engineering, Faculty of Chemical and Food Technology, Slovak University of Technology, Radlinského 2101/9, 812 37 Bratislava, Slovakia
12. Unit of Environmental Engineering, Ben-Gurion University of the Negev, Beer-Sheva, 8410501, Israel
13. Department of Environmental Engineering, Technical University of Denmark, Bygningstorvet, Building 115, 2800 Kgs. Lyngby, Denmark
14. University of Puget Sound, 1500 N. Warner St., Tacoma, WA 98416, United States
15. Department of Chemistry, University of Bath, Bath BA2 7AY, United Kingdom
16. Department of Analytical, Environmental & Forensic Sciences, King's College London, London SE1 9NH, United Kingdom
17. Environmental Pollution Control Laboratory, Chemistry Department, Aristotle University of Thessaloniki, 54124 Thessaloniki, Greece
18. Toxicological Center, Department of Pharmaceutical Sciences, Campus Drie Eiken, University of Antwerp, Universiteitsplein 1, 2610 Antwerp, Belgium
19. IBED, University of Amsterdam, Science Park 904, 1098 XH Amsterdam, The Netherlands
20. Université Paris-Sud, CNRS, AgroParisTech, Université Paris-Saclay, 5 rue Jean Baptiste Clément, Chatenay-Malabry, 92296, France
21. National Institute of Legal Medicine and Forensic Sciences, Rua Manuel Bento de Sousa 3, Lisbon, Portugal
22. NIREAS-International Water Research Center, Department of Civil and Environmental Engineering, University of Cyprus, P.O. Box 20537, 1678, Nicosia, Cyprus
23. University of South Bohemia in Ceske Budejovice, Faculty of Fisheries and Protection of Waters, South Bohemian Research Center of Aquaculture and Biodiversity of Hydrocenoses, Zatisi 728/II, Czech Republic

24. Technische Universität Dresden, Chair of Urban Water Management, 01069 Dresden, Germany
25. Forensic Toxicology, National Institute for Health and Welfare (THL), Mannerheimintie 166, Helsinki, Finland
26. Department of Environmental Sciences, Jožef Stefan Institute, Jamova cesta 39, Ljubljana, Slovenia
27. Rudjer Boskovic Institute, Division for Marine and Environmental Research, Bijenicka 54, Zagreb, Croatia
28. Department of Aquatic Sciences and Assessment, Swedish University of Agricultural Sciences (SLU), Box 7050, SE-750 07 Uppsala, Sweden
29. Institute of Forensic Research, Westerplatte 9, 31-033 Krakow, Poland
30. University of Lisbon, Faculty of Pharmacy, Av. Professor Gama Pinto, 1649-003 Lisbon, Portugal
31. Water and Soil Quality Research Group, Department of Environmental Chemistry, Institute of Environmental Assessment and Water Research (IDAEA-CSIC), Jordi Girona, 18-26, 08034 Barcelona, Spain
32. Department of Pharmacology and Toxicology, University of Iceland, Hofsvallagata 53, Reykjavík, Iceland
33. Institute of Legal Medicine and Core Facility Metabolomics, Medical University of Innsbruck, Innrain 52, 6020 Innsbruck, Austria
34. Queensland Alliance for Environmental Health Sciences (QAEHS), The University of Queensland, 20 Cornwall Street, Woolloongabba, Queensland, 4102, Australia
35. University of Bath, Department of Chemical Engineering, Claverton Down, Bath BA2 7AY, UK
36. Process and Systems Engineering Center (PROSYS), Department of Chemical and Biochemical Engineering, Technical University of Denmark, Building 229, 2800 Kgs. Lyngby, Denmark
37. University of Novi Sad, Faculty of Technical Sciences, Department of Environmental Engineering and Occupational Safety, Trg Dositeja Obradovica 6, Novi Sad 106314, Serbia
38. AGH University of Science and Technology, Department of Coal Chemistry and Environmental Sciences, Al. Mickiewicza 30, Krakow, Poland
39. Department of Chemical Engineering, McGill University, 3610 University, Montreal, Quebec, H3A 0C5, Canada
40. Istituto di Ricerche Farmacologiche Mario Negri IRCCS, Via Giuseppe La Masa, 19, 20156 Milan, Italy
41. Swiss Federal Institute of Aquatic Science and Technology, Eawag, Urban Water Management, Überlandstrasse 133, 8600 Dübendorf, Switzerland

Table S1. Population served by each wastewater treatment plant (WWTP). Countries are defined by a two-letter abbreviation following the ISO 3166-1 alpha-2 code.

		Population of the city under investigation ①	Estimated population in WWTP catchment							Coverage during last year participation (%) ②
City	country		2011	2012	2013	2014	2015	2016	2017	
Innsbruck	AT	130 894 (W 2016)						262 780	261 503	200
Klosterneuburg	AT	26 750 (W 2016)					30 000			112
Adelaide (4)	AU	1 333 927 (W 2017)							1 133 475	85
Adelaide BA	AU	"							689 802	
Adelaide BH	AU	"							91 394	
Adelaide G	AU	"							207 154	
Adelaide CB	AU	"							145 125	
Canberra	AU	410 301 (W 2017)				338 888	338 888		390 706	95
Toowoomba	AU	135 631 (W 2017)				125 000	125 000		125 000	92
Sarajevo	BA	C: 395 133 M: 688 437 (W)			130 000			150 000		38 C
Antwerp (2)	BE	520 504 (W 2017)		344 094	344 094	344 094	344 094	344 094		66
Antwerp D	BE	"		213 876	213 876	213 876	213 876	213 876		
Antwerp Z	BE	"	117 200	130 218	130 218	130 218	130 218	130 218	130 218	
Boom	BE	17 970 (W 2018)							30 600	170
Brussels	BE	1 196 831 (E 2015)	1 027 300	953 987	953 987		953 987	953 987	953 987	80
Geraardsbergen	BE	33 403 (W 2018)			29 047	29 047	29 047			87
Koksijde	BE	21 957 (W 2018)			78 441		78 441	78 441	78 441	357
Merchtem	BE	16 294 (W 2018)							16 200	99
Ninove	BE	37 289 (W 2012)			36 179	36 179	36 179			97

Oostende	BE	70 813 (E 2015)				159 000	159 000	159 000	225
Ruisbroek	BE	34 038 (W 2018)						36 000	106
Sofia	BG	1 549 659 (E 2017)					1 200 000		77
Granby	CA	63 433 (W 2011)			55 255	55 255			87
Montreal	CA	1 704 694 (W 2016)			1 958 257	1 958 257			115
Basel	CH	C: 175 940 M: 319 220 (W 2017, E 2014)	260 000	260 000	260 000	260 000	260 000	260 000	81 M
Berne	CH	C: 142 656 M: 406 900 (W 2018)	206 655	206 655	206 655	206 655	206 655	206 655	51 M
Biel	CH	53 031 (E 2014)			82 285				155
Chur	CH	34 880 (W 2008)			52 800	52 800			151
Geneva	CH	198 979 (W 2016)	410 486	410 486	417 200	417 200	417 200	417 200	210
Lausanne	CH	229 425 (E 2014)			220 000	220 000			96
Lugano	CH	83 727 (E 2014)			103 561	103 561	103 561		124
Luzern	CH	159189 (E 2014)			174 800	174 800			110
Neuchatel	CH	33 772 (W 2016)			50 000	50 000			148
Sion	CH	33 999 (W 2016)			45 000	45 000			132
St.Gallen (2)	CH	75 481 (W 2016)	89 000	89 000					118
St.Gallen H	CH	"	52 000	52 000	52 000	52 000	52 000	52 000	
St.Gallen A	CH	"	37 000	37 000					
Winterthur	CH	105 676 (E2014)			125 000	125 000			118
Zurich	CH	402 762 (W 2016)	410 000	410 000	410 000	410 000	410 000	410 000	102
Bogota	CO	8 080 734 (W 2017)				2 500 000			31
Medellin	CO	2 441 123 (W 2013)				600 000			25
Nicosia	CY	234 200 (E 2009)		28 000		28 000	28 000	28 000	12
Limassol	CY	185 100 (E 2009)		272 000		120 000	120 000	120 000	65
Brno	CZ	377 028 (E 2015)						445 000	118

Budweis	CZ	93 285 (W 2015)	112 000	112 000	110 300		110 300	110 300	110 300	118
Prague	CZ	1 297 000 (W 2018)		1 300 000	1 300 000					100
Chemnitz	DE	246 855 (W 2017)							239 402	97
Berlin (4)	DE	3 469 849 (E 2015)				3 840 000	3 840 000		3 993 500	115
Berlin M	DE	"				290 000	290 000		333 500	
Berlin R	DE	"				1 300 000	1 300 000		1 560 000	
Berlin S	DE	"				750 000	750 000		750 000	
Berlin W	DE	"				1 500 000	1 500 000		1 350 000	
Dortmund	DE	580 511 (E 2015)			371 788	371 788	371 788	371 788	371 788	64
Dülmen	DE	46 523 (W 2016)			34 495	34 495	34 495	34 046	44 428	95
Dresden	DE	536 308 E 2015)			593 050	593 050	593 050	669 675	669 675	125
Erfurt	DE	212 988 (W 2017)							213 977	100
Munich (2)	DE	1 429 584 (E 2015)						1 739 040	1 826 704	128
Munich G	DE	"				1 000 000	1 000 000	1 043 424	1 181 985	
Munich M	DE	"						695 616	644 719	
Nuremberg	DE	515 201 (W 2017)							453 914	88
Hamburg (2)	DE	1 762 791 (E 2015)							2 050 000	116
Hamburg N	DE	"							630 000	
Hamburg S	DE	"							1 420 000	
Hannover (2)	DE	535 061 (W 2017)							740 000	138
Hannover H	DE	"							325 000	
Hannover G	DE	"							415 000	
Frankfurt (3)	DE	717 624 (E 2015)							1 118 389	156
Frankfurt N	DE	"							357 230	
Frankfurt G	DE	"							520 485	
Frankfurt S	DE	"							240 674	

Magdeburg	DE	232 306 (E 2015)							278 236	120
Mainz	DE	215 110 (W 2017)							200 344	93
Rostock	DE	208 409 (W 2017)							237 316	114
Saarbrucken (2)	DE	180 966 (W 2017)							203 164	112
Saarbrucken Br	DE	"							81 399	
Saarbrucken Bu	DE	"							121 765	
Stuttgart	DE	632 743 (W 2017)							602 000	95
Copenhagen	DK	763 908 (W 2018)			531 000	531 000	531 000			70
Barcelona	ES	C: 1 621 090 M: 3 624 554 (E 2016)	1 162 000	1 162 000	1 162 000	1 150 874	1 150 874	1 149 722	1 154 819	71 C
Castellon	ES	170 990 (W 2016)	170 600	204 878	204 878	180 690	180 690	171 669	171 669	100
Molina de Segura	ES	69 614 (W 2016)					70 709	70 709		102
Santiago	ES	95 966 (E 2016)	136 500	136 500	136 500	136 500	136 500	136 500	136 500	142
Valencia (3)	ES	C: 809 267 M: 1 388 368 (E 2016)			1 357 952	1 357 952	1 310 859	1 310 859	1 191 210	86 M
Valencia P1	ES	"			322 926	322 926	322 926	322 926	388 657	
Valencia P2	ES	"			820 026	820 026	820 026	820 026	652 495	
Valencia QB	ES	"			215 000	215 000	167 907	167 907	150 058	
Espoo	FI	269 802 (E 2015)				300 000	300 000	300 000	300 000	111
Helsinki	FI	1 122 101 (E 2015)	780 000	780 000	780 000	800 000	800 000	800 000	800 000	71
Joensuu	FI	75 652 (W 2017)				75 000		81 849		108
Jyväskylä	FI	137 368 (E 2015)				135 000		135 000		98
Kotka	FI	53 730 (W 2017)				72 500		72 500		135
Kuopio	FI	116 921 (E 2015)				80 000		85 588		73
Lahti	FI	118 743 (E 2015)				63 000		63 000		53
Lappeenranta	FI	72 685 (W 2017)				60 000		60 000		83
Oulu	FI	198 525 (E 2015)				195 000		174 500		88

Rovaniemi	FI	62 037 (W 2017)				49 000		49 000		79
Savonlinna	FI	34 829 (W 2017)				28 000		28 000		80
Tampere	FI	225 118 (E 2015)				200 000	200 000	200 000	200 000	89
Turku	FI	185 908 (E 2015)	275 000	275 000	275 000	285 000	285 000	285 000	300 000	161
Vaasa	FI	66 876 (W 2017)				67 000		69 252		104
Bordeaux (2)	FR	753 601 (E 2014)							481 638	64
Bordeaux 1	FR	"						254 338	254 338	
Bordeaux 2	FR	"							227 300	
Fort-de-France D	FR	160 498 (E 2014)				16 500				10
Fort-de-France P	FR	"				19 000	19 000		19 000	
Paris G	FR	C: 2 206 488 (W 2018)<br>M: 6 754 282 (E 2014)		245 500						11 C
Paris SC	FR	"	774 600		1 004 000	1 004 000	1 004 000	1 004 000	461 866	21
Paris SE	FR	"							85 263	4
Bristol	GB	459 300 (W 2017)				886 650	886 650	886 650	886 650	193
London	GB	8 730 803 (E 2017)	3 400 000		3 400 000	3 400 000	3 400 000	3 400 000		39
Athens	GR	3 090 508 (W 2011)			3 700 000	3 700 000	3 700 000	3 700 000	3 700 000	120
Mytilene	GR	37 890 (W 2011)					26 000			69
Thessaloniki	GR	C: 325 182 M: 788 952 (W 2011)				850 000	850 000			108 M
Zagreb	HR	801 349 (E)	650 000	650 000	650 000	650 000	650 000	650 000	650 000	81
Reykjavik (2)	IS	127 220 (W 2018)						186 000	184 292	145
Reykjavik N	IS	"					93 000	93 000	97 168	
Reykjavik S	IS	"						93 000	87 124	
Tel-Aviv	IL	443 939 (W 2017)						89 358		20
Milan	IT	2 640 159 (E 2018)	1 250 000	1 100 000	1 149 477	1 122 501	1 122 501	1 140 000	1 080 000	41
Bari	IT	323 370 (W 2018)							459 650	142

Bologna	IT	389 261 (W 2018)							500 000	128
Gorizia	IT	34 411 (W 2018)							34 844	101
Potenza	IT	67 211 (W 2018)							90 000	134
Palermo	IT	668 405 (W 2018)							360 000	54
Busan	KR	3 506 103 (W 2018)					1 164 950			33
Bendern	LI	37 800 (W 2017)				37 000	37 000			98
Vilnius	LT	536 631 (W 2018)							545 280	102
Malta	MT	475 700 (W 2018)					296 297			62
Amsterdam	NL	810 938 (E 2014)	694 800	769 000	769 000	769 000	769 000		769 000	95
Eindhoven	NL	220 920 (E 2014)	448 700	450 300	450 300	450 300	450 300	450 300	450 300	204
Utrecht	NL	328 164 (E 2014)	297 000	300 000	300 000	300 000	300 000	300 000	300 000	91
Nieuwegein Ijssel.	NL	62 235 (W 2017)							96 000	154
Oudewater	NL	10 108 (W 2017)							10 110	100
Auckland (2)	NZ	1 534 700 (W 2017)					1 332 000			87
Auckland M	NZ	"					1 100 000			
Auckland R	NZ	"					232 000			
Oslo	NO	673 469 (W 2018)	557 000	557 000	576 000	580 639	580 639	580 639	580 639	86
Krakow (2)	PL	766 739 (W 2017)						930 000	930 000	121
Krakow K	PL	"						250 000	250 000	
Krakow P	PL	"						680 000	680 000	
Almada	PT	174 030 (W 2011)				138 685	138 685	138 685	138 685	80
Lisbon	PT	505 526 (W 2017)			426 964		426 964	426 964	426 964	84
Porto	PT	287 591 (W)					150 000	150 000	150 000	52
Alba Iulia	RO	74 283 (E 2015)						63 000		85
Cluj Napoca	RO	321 916 (E 2015)			350 000			360 000		112
Beograd	RS	1 166 763 (W 2011)			284 347			500 000		43

Novi Sad	RS	277 522 (W 2011)			321 282					116
Gothenburg	SE	520 374 (E 2011)		664 441	664 441					128
Stockholm (2)	SE	1 579 896 (E 2011)						784 000		50
Stockholm H	SE	"	315 000					326 000		
Stockholm S	SE	"						458 000		
Umeå	SE	116 465 (E 2011)	115 800	115 800	115 800					99
Ljubljana	SI	288 307 (E)							266 131	92
Bratislava (2)	SK	422 932 (E)			440 000		470 000	470 000	600 000	142
Bratislava C	SK	"			320 000		350 000	350 000	450 000	
Bratislava P	SK	"			120 000		120 000	120 000	150 000	
Piestany	SK	27 777 (W 2016)			30 000		30 000	30 000	30 000	108
Uzhhorod	UA	115 163 (W 2016)						116 200		101
Seattle	US	608 660 (W 2010)					893 000	893 000	893 000	147

① Population of entire city/region. C: city. M: metropolitan, greater region. (E, W): Eurostat, Wikipedia (year).
② For cases where WTP's catchment area is bigger than the actual city (i.e. covering suburbs, see Ruisbroek_BE) coverages are > 100%. Coverage close to 100% does not imply a perfect alignment between city boundary and WTP catchment area. For cases where coverage is far below 100% is very likely the existance of supplementary WTP in the city that have not participated in the study

Table S2. Date of the beginning of the monitoting campaign and sampling mode used to collect wastewater samples.

		Monitoring Date ①/Sampling Mode ②													
City	Country	2011		2012		2013		2014		2015		2016		2017	
Innsbruck	AT											29.3	υ	4.4	υ
Klosterneuburg	AT									10.6	υ				
Adelaide BA	AU													2.4	nr
Adelaide BH	AU													2.4	nr
Adelaide G	AU													2.4	nr
Adelaide CB	AU													2.4	nr
Canberra	AU							12.3	nr	18.3	nr			5.8	nr
Toowoomba	AU							12.3	υ	17.3	υ			4.8	υ
Sarajevo	BA					6.3	t					25.5	t		
Antwerp D	BE			17.4	t	6.3	t	4.4	t	18.3	t	28.9	t		
Antwerp Z	BE	9.3	t	17.4	t	6.3	t	3.4	t	18.3	t	28.9	t	19.4	t
Boom	BE													17.5	t
Brussels	BE	9.3	υ	17.4	υ	6.3	υ			18.3	υ	28.9	υ	20.4	υ
Geraardsbergen	BE					7.3	t	19.3	t	11.3	t				
Koksijde	BE					21.3	t			11.3	t	19.5	t	19.4	t
Merchtem	BE													16.5	t
Ninove	BE					6.3	t	19.3	t	11.3	t				
Oostende	BE									11.3	t	19.5	t	19.4	t
Ruisbroek	BE													17.5	t
Sofia	BG											24.5	t		
Granby	CA							11.3	t	17.3	t				
Montreal	CA							11.3	υ	17.3	υ				

Basel	CH			17.4	υ	6.3	υ	18.3	υ	18.3	υ	16.3	υ	22.3	υ
Berne	CH			17.4	υ	6.3	υ	18.3	υ	18.3	υ	16.3	υ	22.3	υ
Biel	CH							18.3	υ						
Chur	CH							18.3	ł	18.3	ł				
Geneva	CH			17.4	υ	6.3	υ	18.3	υ	18.3	υ	16.3	υ	22.3	υ
Lausanne	CH							18.3	υ	18.3	υ				
Lugano	CH							18.3	t	25.3	t	16.3	t		
Luzern	CH							18.3	υ	18.3	υ				
Neuchatel	CH							18.3	υ	18.3	υ				
Sion	CH							18.3	t	18.3	t				
St.Gallen H	CH			18.4	υ	6.3	υ	18.3	υ	18.3	υ	16.3	υ	22.3	υ
St.Gallen A	CH			17.4	υ	6.3	υ								
Winterthur	CH							18.3	υ	18.3	υ				
Zurich	CH			17.4	υ	6.3	υ	18.3	υ	18.3	υ	16.3	υ	22.3	υ
Bogota	CO									10.3	υ				
Medellin	CO									27.6	t				
Nicosia	CY					21.3	υ			4.4	υ	16.3	υ	22.3	υ
Limassol	CY					21.3	υ			4.4	t	16.3	t	22.3	t
Brno	CZ													5.4	υ
Budweis	CZ	9.3	t	17.4	t	6.3	t			18.3	t			5.4	t
Prague	CZ			17.4	ł	6.3	t								
Chemnitz	DE													13.6	t
Berlin M	DE							11.3	υ	19.3	υ			29.3	υ
Berlin R	DE							11.3	υ	19.3	υ			29.3	υ
Berlin S	DE							11.3	υ	19.3	υ			29.3	υ
Berlin W	DE							11.3	υ	19.3	υ			29.3	υ

Dortmund	DE					13.3	υ	12.3	υ	18.3	υ	5.4	υ	5.4	υ
Dülmen	DE					6.3	t	12.3	t	18.3	t	5.4	t	5.4	t
Dresden	DE					6.3	υ	11.3	υ	18.3	υ	5.4	υ	5.4	υ
Erfurt	DE													5.4	nr
Munich G	DE							12.3	t	20.3	t	5.4	t	5.4	t
Munich M	DE											5.4	t	5.4	t
Nuremberg	DE													5.4	nr
Hamburg N	DE													5.4	ŧ
Hamburg S	DE													5.4	ŧ
Hannover H	DE													13.6	t
Hannover G	DE													13.6	t
Frankfurt N	DE													5.4	t
Frankfurt G	DE													5.4	t
Frankfurt S	DE													5.4	t
Magdeburg	DE													5.4	ŧ
Mainz	DE													13.6	t
Rostock	DE													13.6	nr
Saarbrucken Br	DE													13.6	nr
Saarbrucken Bu	DE													13.6	nr
Stuttgart	DE													5.4	nr
Copenhagen	DK					6.3	υ	11.3	υ	10.3	υ				
Barcelona	ES	16.3	t	17.4	t	6.3	t	18.3	t	11.3	t	9.3	t	9.3	t
Castellon	ES	9.3	ŧ	17.4	ŧ	6.3	t	11.3	t	25.3	t	8.3	t	6.4	t
Molina de Segura	ES									15.4	t	6.4	t		
Santiago	ES	9.3	t	17.4	t	6.3	t	11.3	t	4.3	t	19.4	t	27.3	t
Valencia P1	ES					6.3	t	11.3	t	4.3	t	2.3	t	2.5	t

Valencia P2	ES					6.3	t	11.3	t	4.3	t	2.3	t	2.5	t
Valencia QB	ES					6.3	t	11.3	t	4.3	t	2.3	t	2.5	t
Espoo	FI							11.3	υ	11.3	υ	16.3	υ	22.3	υ
Helsinki	FI	9.3	υ	17.4	υ	6.3	υ	11.3	υ	11.3	υ	16.3	υ	22.3	υ
Joensuu	FI							18.3	t			16.3	t		
Jyväskylä	FI							11.3	υ			16.3	υ		
Kotka	FI							11.3	υ			16.3	υ		
Kuopio	FI							11.3	υ			16.3	υ		
Lahti	FI							25.3	υ			16.3	υ		
Lappeenranta	FI							11.3	t			16.3	t		
Oulu	FI							11.3	t			16.3	t		
Rovaniemi	FI							11.3	t			16.3	t		
Savonlinna	FI							11.3	t			16.3	t		
Tampere	FI							11.3	υ	11.3	υ	16.3	υ	22.3	υ
Turku	FI	9.3	υ	17.4	υ	6.3	υ	11.3	υ	11.3	υ	16.3	υ	22.3	υ
Vaasa	FI							11.3	υ			16.3	υ		
Bordeaunr 1	FR											26.9	υ	15.6	υ
Bordeaunr 2	FR													15.6	υ
Fort-de-France D	FR							27.3	ƚ						
Fort-de-France P	FR							27.3	ƚ	28.5	ƚ			1.7	ƚ
Paris G	FR			21.4	υ										
Paris SC	FR	9.3	υ			6.3	υ	11.3	υ	23.3	υ	31.3	υ	29.4	υ
Paris SE	FR													19.4	ƚ
Bristol	GB							11.3	t	10.3	t	7.3	t	22.3	t
London	GB	9.3	t			6.3	t	11.3	t	11.3	t	7.3	t		
Athens	GR					6.3	ƚ	11.3	ƚ	4.3	ƚ	16.3	ƚ	3.8	ƚ

Mytilene	GR									11.2	nr				
Thessaloniki	GR							11.3	nr	28.5	nr				
Zagreb	HR	9.3	t	17.4	t	6.3	t	11.3	t	17.3	t	9.3	t	21.3	t
Reykjavik N	IS									27.6	t	16.3	t	22.3	t
Reykjavik S	IS											16.3	t	22.3	t
Tel-Aviv	IL											24.3	t		
Milan	IT	9.3	υ	17.4	υ	6.3	υ	11.3	υ	10.3	υ	16.3	υ	28.3	υ
Bari	IT													15.5	t
Bologna	IT													8.5	υ
Gorizia	IT													8.5	t
Potenza	IT													15.5	t
Palermo	IT													15.5	t
Busan	KR									18.3	t				
Bendern	LI							18.3	t	18.3	t				
Vilnius	LT													22.3	t
Malta	MT									18.3	nr				
Amsterdam	NL	9.3	υ	17.4	υ	6.3	υ	13.3	υ	4.3	υ			19.4	υ
Eindhoven	NL	9.3	υ	17.4	υ	6.3	υ	11.3	υ	4.3	υ	9.3	υ	19.4	υ
Utrecht	NL	10.3	υ	17.4	υ	6.3	υ	11.3	υ	4.3	υ	9.3	υ	19.4	υ
Nieuwegein Ijssel.	NL													19.4	nr
Oudewater	NL													19.4	nr
Auckland M	NZ									4.3	t				
Auckland R	NZ									4.3	υ				
Oslo	NO	9.3	υ	17.4	υ	6.3	υ	11.3	υ	11.3	υ	11.3	υ	26.5	υ
Krakow K	PL											3.4	t	12.2	t
Krakow P	PL											3.4	t	12.2	t

Almada	PT							11.3	ʈ	24.3	ʈ	12.4	ʈ	22.3	ʈ
Lisbon	PT					6.3	ʈ			26.3	ʈ	12.4	ʈ	22.3	ʈ
Porto	PT									23.4	nr	20.4	nr	3.5	nr
Alba Iulia	RO											25.5	nr		
Cluj Napoca	RO					6.3	nr					24.5	nr		
Beograd	RS					17.3	ʈ					25.5	nr		
Novi Sad	RS					6.3	ʈ								
Gothenburg	SE			17.4	ɫ	6.3	ɫ								
Stockholm H	SE	9.3	nr									11.5	nr		
Stockholm S	SE											11.5	nr		
Umeå	SE	9.3	ʋ	17.4	ʋ	6.3	ʋ								
Ljubljana	SI													21.3	ʈ
Bratislava C	SK					6.3	ɫ			11.3	ɫ	9.3	ɫ	24.3	ɫ
Bratislava P	SK					6.3	ɫ			17.3	ɫ	9.3	ɫ	24.3	ɫ
Piestany	SK					6.3	ʈ			11.3	ʈ	9.3	ʈ	21.3	ʈ
Uzhhorod	UA											26.5	nr		
Seattle	US									24.3	nr	14.6	nr	15.6	nr

① Monitoring day: first day of the monitoring week (day.month)

② Sampling mode: ʈ time proportional, ʋ volume proportional, ɫ flow proportional, nr: not reported

Table S3. Biomarkers used, excretion rates, correction factors and standardized average doses used for back-calculation of consumption of cocaine, amphetamine, methamphetamine and MDMA combined.

Compound	Drug Residue	Excretion rate (%)	Correction Factor	Average dose (mg)
Cocaine	benzoylecgonine	29	3.59[1]	100[3]
Amphetamine	amphetamine	36	2.77[2]	50[4]
Methamphetamine	methamphetamine	41	2.44[2]	50[4]
MDMA	MDMA	22.5	4.4[2]	100[3]

[1] Castiglioni S, Bijlsma L, Covaci A, Emke E, Hernández F, Reid M et al., Zuccato E. Evaluation of Uncertainties Associated with the Determination of Community Drug Use through the Measurement of Sewage Drug Biomarkers. Environmental Science & Technology. 2013;47:1452-60

[2] Gracia-Lor E, Zuccato E, Castiglioni S. Refining correction factors for back-calculation of illicit drug use. Science of the Total Environment. 2016;573:1648-59

[3] Zuccato E, Chiabrando C, Castiglioni S, Bagnati R, Fanelli R. Estimating community drug abuse by wastewater analysis. Environmental Health Perspectives. 2008;116:1027-32

[4] http://www.emcdda.europa.eu/drug-profiles, archived at http://www.webcitation.org/73dJQl1ua on 2/11/2018

Uncertainties related to WBE

Uncertainties related to the mass of drug residues that enter the sewer system through toilet flushes are listed in the left column of Table S3. Uncertainties originating from sampling and chemical analysis can be quantified and minimized (*). For flow measurements plausibility checks are performed to exclude gross errors ($^{+}$). For these aspects, all partners stick to the best practice protocol developed by SCORE[1]. Other aspects such as losses from sewers, in-sewer transformation and population size cannot be addressed in-depth in such large-scale screening studies (°). In addition, the uncertainties in the right column of Table S3 affect the comparison of absolute numbers of doses consumed. These aspects are relevant when comparing WBE to consumption estimates obtained with other methods (global population survey) and, in the context of this study, only for the presentation of “combined doses”.

Table S4. List of uncertainties and their characterization into *random* or **systematic**.

Uncertainties relevant for estimation of population-normalized …			
… drug residue mass in sewers	refs	… number of doses consumed	refs
**losses from sewers (exfiltration)°**	2	**excretion rates**	13, 17
**in-sewer transformation°**	3-9	***standard doses***	-
sample collection*	10	***purity***	-
**flow measurements**$^{+}$	2		
*chemical analyses**	11		
Population size°	12-15		
annual average	16		
drug residues not discharged to sewers	-		

[1] European Monitoring Centre for Drugs and Drug Addiction, “Common protocol of action for monitoring illicit drugs in wastewater”, available at: https://goo.gl/seR7Ke

[2] Ort C, van Nuijs ALN, Berset JD, Bijlsma L, Castiglioni S, Covaci A, et al., Thomas KV. Spatial differences and temporal changes in illicit drug use in Europe quantified by wastewater analysis. Addiction. 2014;108(9):1338-52

[3] Li J, Gao J, Thai PK, Sun X, Mueller JF, Yuan Z, Jiang G. Stability of Illicit Drugs as Biomarkers in Sewers: From Lab to Reality. Environmental Science & Technology. 2018;52(3):1561-70

[4] McCall AK, Palmitessa R, Blumensaat F, Morgenroth E, Ort C. Modeling in-sewer transformations at catchment scale – implications on drug consumption estimates in wastewater-based epidemiology. Water Research. 2017;122:655–68

[5] McCall AK, Scheidegger A, Madry MM, Steuer AE, Weissbrodt DG, Vanrolleghem PA, et al., Ort, C. Influence of different sewer biofilms on transformation rates of drugs. Environmental Science & Technology. 2016;50:13351–60

[6] Ramin P, Brock AL, Polesel F, Causanilles A, Emke E, de Voogt P, Plósz BG. Transformation and sorption of illicit drug biomarkers in sewer systems: understanding the role of suspended solids in raw wastewater. Environmental Science & Technology. 2016;50:13397–408

[7] Ramin P, Polesel F, Brock AL, Plósz BG. The impact of temperature on the transformation of illicit drug biomarkers in wastewater. Science of the Total Environment. 2018;644:1612–16

[8] Senta I, Krizman I, Ahel M, Terzic S. Assessment of stability of drug biomarkers in municipal wastewater as a factor influencing the estimation of drug consumption using sewage epidemiology. Science of the Total Environment. 2014;487:659–65

[9] Thai PK, Jiang G, Gernjak W, Yuan Z, Lai FY, Mueller JF. Effects of sewer conditions on the degradation of selected illicit drug residues in wastewater. Water Research. 2014;48:538–47

[10] Ort C, Lawrence MG, Rieckermann J, Joss A. Monitoring Pharmaceuticals and Personal Care Products (PPCPs) and Illicit Drugs in Wastewater Systems: Are Your Conclusions Valid? A Critical Review. Environmental Science & Technology. 2010;44(16):6024–35

[11] van Nuijs ALN, Been F, Lai FY, Andres-Costa MJ, Barron L, Baz-Lomba JA, et al., Ort C. Multi-year interlaboratory exercises for the analysis of illicit drugs and metabolites in wastewater: development of a quality control system. Trends in Analytical Chemistry. 2018;103:34-43

[12] O'Brien JW, Thai P, Ort C, Scheidegger A, Eaglesham G, Carter S, Lai FY, Mueller JF. A model to estimate the population contributing to the wastewater using samples collected on census day. Environmental Science & Technology. 2014;48(1):517-25

[13] Castiglioni S, Bijlsma L, Covaci A, Emke E, Hernández F, Reid M, et al., Zuccato E. Evaluation of uncertainties associated with the determination of community drug consumption through the measurement of sewage biomarkers. Environmental Science & Technology. 2013;47(3):1452–60

[14] O'Brien JW, Banks APW, Novic AJ, Mueller JF, Jiang G, Ort C, et al., Thai P.K. Impact of in-Sewer Degradation of Pharmaceutical and Personal Care Products (PPCPs) Population Markers on a Population Model. Environmental Science & Technology. 2017;51(7):3816-23

[15] Lai FY, Anuj S, Bruno R, Carter S, Gartner C, Hall W, et al., Ort C. Systematic and day-to-day effects of chemical-derived population estimates on wastewater-based drug epidemiology. Environmental Science & Technology. 2015;49:999-1008

[16] Ort C, Eppler JM, Scheidegger A, Rieckermann J, Kinzig M, Sörgel F. Challenges of surveying wastewater drug loads in small populations and generalizable aspects on optimizing monitoring design. Addiction. 2014;109(3):472-81

[17] Gracia-Lor E, Zuccato E, Castiglioni S. Refining correction factors for back-calculation of illicit drug use. Science of the Total Environment. 2016;573:1648-59

Cannabis (THC-COOH): results and discussion

Population-normalized mass loads of THC-COOH are only available for a limited number of locations (see Appendix S3, cities with either a value or <LOQ). The highest loads were observed in Amsterdam, Barcelona and Paris SC and, among the locations sampled outside Europe, in Seattle and Fort-de-France (Appendix S3). Furthermore, the high loads observed in Novi Sad are noteworthy, yet the sampling and analysis in this location was performed only on one occasion (i.e. 2013) and, therefore, results have to be interpreted with caution. Interestingly, intra-country differences were observed for those countries where multiple cities were sampled and analysed: in general, larger cities showed higher loads of THC-COOH.

In terms of temporal variations, most locations showed a rather stable cannabis use across years. However, some cities showed a clear increasing or decreasing trend. In Barcelona and Zagreb, mass loads rose significantly between 2011 and 2017 with a factor of 3.5 to 4. Two out of the three studied catchments in Paris (G and SE) showed high population-normalized loads of THC-COOH during the only year they participated in the study. However, the trends in Paris SC, which was monitored 5 years, showed a 40% decrease between 2011 and 2017.

In contrast to cocaine and amphetamine-type stimulants, the use of cannabis, calculated from measurements of THC-COOH, does not show any clear geographical pattern in Europe and only some geographical trends are in agreement with numbers obtained from prevalence and production indicators. As an example, the highest THC-COOH loads were found in cities from NL, a country with a liberal drug policy on soft drugs[1], and from ES, the major point of entry of cannabis resin produced in Morocco[2]. On a temporal analysis, the stable loads found in most of the locations sampled 5 years or more agree with the most recent surveys on cannabis use, which also point to a stable consumption of this drug in most countries[2].

[1] Bijlsma L., Emke E., Hernández F., de Voogt P. Investigation of drugs of abuse and relevant metabolites in Dutch sewage water by liquid chromatography coupled to high resolution mass spectrometry. Chemosphere 2012;89: 1399–406

[2] European Monitoring Centre for Drugs and Drug Addiction 2017. European Drug Report 2017: Trends and Developments, Publications Office of the European Union, Luxembourg.
