# Supplementary material for: Spatio‐temporal assessment of illicit drug use at large scale: evidence from 7 years of international wastewater monitoring
Source: Addiction. 2019 Oct 23;115(1):109–20. doi: 10.1111/add.14767 (PMC6973045; doi:10.1111/add.14767)
Supplement: Supplementary file 2 — Appendix S2 Population‐normalized loads for benzoylecgonine, amphetamine, methamphetamine, MDMA and THC‐COOH and population‐normalized number of combined doses. In addition, an open online repository available at https://doi.org/10.25678/000172 compiles load results. [file ADD-115-109-s002.pdf]

Appendix 2

Spatio-temporal assessment of illicit drug use at large scale: evidence from seven years of international wastewater monitoring

Authors:

Iria González-Mariño[1,2,*], Jose Antonio Baz-Lomba[3]
Nikiforos A. Alygizakis[4], Maria Jesús Andrés-Costa[5], Richard Bade[6], Leon P. Barron[7], Frederic Been[8], Jean-Daniel Berset[9], Lubertus Bijlsma[10], Igor Bodík[11], Asher Brenner[12], Andreas L. Brock[13], Daniel A. Burgard[14], Erika Castrignanò[15,16], Christophoros E. Christophoridis[17], Adrian Covaci[18], Pim de Voogt[19], Damien A. Devault[20], Mário J. Dias[21], Erik Emke[8], Despo Fatta-Kassinos[22], Ganna Fedorova[23], Konstantinos Fytianos[17], Cobus Gerber[6], Roman Grabic[23], Stefan Grüner[24], Teemu Gunnar[25], Evroula Hapeshi[22], Ester Heath[26], Björn Helm[24], Félix Hernández[10], Aino Kankaanpaa[25], Sara Karolak[20], Barbara Kasprzyk-Hordern[15], Ivona Krizman-Matasic[27], Foon Yin Lai[28], Wojciech Lechowicz[29], Alvaro Lopes[30], Miren López de Alda[31], Ester López-García[31], Arndís S.C. Löve[32], Nicola Mastroianni[31], Gillian L. McEneff[7], Rosa Montes[1], Kelly Munro[7], Thomas Nefau[20], Herbert Oberacher[33], Jake W. O'Brien[34], Kristin Olafsdottir[32], Yolanda Picó[5], Benedek G. Plósz [13,35], Fabio Polesel[13], Cristina Postigo[31], José Benito Quintana[1], Pedram Ramin[13,36], Malcolm J. Reid[3], Jack Rice[15], Rosario Rodil[1], Ivan Senta[27], Susana M. Simões[21], Maja M. Sremacki[37], Katarzyna Styszko[38], Senka Terzic[27], Nikolaos S. Thomaidis[4], Kevin V. Thomas[3,34], Ben J. Tscharke[34], Alexander L.N. van Nuijs[18], Viviane Yargeau[39], Ettore Zuccato[40], Sara Castiglioni[40,*], Christoph Ort[41].

*Corresponding authors:
Iria González-Mariño: Tel: +34 881 816 035; e-mail: iria.gonzalez@usc.es, iriagonzalez@usal.es
Sara Castiglioni: Tel: +39 02 39014776; e-mail: sara.castiglioni@marionegri.it

1. Institute for Food Analysis and Research, Department of Analytical Chemistry, Universidade de Santiago de Compostela, 15782 Santiago de Compostela, Spain
2. Faculty of Chemical Sciences, Department of Analytical Chemistry, Nutrition and Bromatology, University of Salamanca, 37008 Salamanca, Spain
3. Norwegian Institute for Water Research (NIVA), Gaustadalléen 21, NO-0349 Oslo, Norway
4. National and Kapodistrian University of Athens, Department of Chemistry, Laboratory of Analytical Chemistry, 15771 Athens, Greece
5. Food and Environmental Safety Research Group (SAMA-UV) - CIDE (CSIC-UV-GV), University of Valencia, Moncada-Naquera Road Km 4,3, 46113 Moncada, Spain
6. School of Pharmacy and Medical Sciences, University of South Australia, GPO Box 2471, Adelaide, South Australia 5001, Australia
7. King's Forensics, School of Population Health & Environmental Sciences, King's College London, London SE1 9NH, United Kingdom
8. KWR Watercycle Research Institute, P.O. Box 1072, 3430 BB, Nieuwegein, The Netherlands
9. University of Bern, Institute of Plant Sciences, Altenbergrain 21, 3013 Bern, Switzerland
10. Research Institute for Pesticides and Water, University Jaume I, Avda. Sos Baynat s/n, E-12071 Castellón, Spain
11. Department of Environmental Engineering, Faculty of Chemical and Food Technology, Slovak University of Technology, Radlinského 2101/9, 812 37 Bratislava, Slovakia
12. Unit of Environmental Engineering, Ben-Gurion University of the Negev, Beer-Sheva, 8410501, Israel
13. Department of Environmental Engineering, Technical University of Denmark, Bygningstorvet, Building 115, 2800 Kgs. Lyngby, Denmark
14. University of Puget Sound, 1500 N. Warner St., Tacoma, WA 98416, United States
15. Department of Chemistry, University of Bath, Bath BA2 7AY, United Kingdom
16. Department of Analytical, Environmental & Forensic Sciences, King's College London, London SE1 9NH, United Kingdom
17. Environmental Pollution Control Laboratory, Chemistry Department, Aristotle University of Thessaloniki, 54124 Thessaloniki, Greece
18. Toxicological Center, Department of Pharmaceutical Sciences, Campus Drie Eiken, University of Antwerp, Universiteitsplein 1, 2610 Antwerp, Belgium
19. IBED, University of Amsterdam, Science Park 904, 1098 XH Amsterdam, The Netherlands
20. Université Paris-Sud, CNRS, AgroParisTech, Université Paris-Saclay, 5 rue Jean Baptiste Clément, Chatenay-Malabry, 92296, France
21. National Institute of Legal Medicine and Forensic Sciences, Rua Manuel Bento de Sousa 3, Lisbon, Portugal
22. NIREAS-International Water Research Center, Department of Civil and Environmental Engineering, University of Cyprus, P.O. Box 20537, 1678, Nicosia, Cyprus
23. University of South Bohemia in Ceske Budejovice, Faculty of Fisheries and Protection of Waters, South Bohemian Research Center of Aquaculture and Biodiversity of Hydrocenoses, Zatisi 728/II, Czech Republic

24. Technische Universität Dresden, Chair of Urban Water Management, 01069 Dresden, Germany
25. Forensic Toxicology, National Institute for Health and Welfare (THL), Mannerheimintie 166, Helsinki, Finland
26. Department of Environmental Sciences, Jožef Stefan Institute, Jamova cesta 39, Ljubljana, Slovenia
27. Rudjer Boskovic Institute, Division for Marine and Environmental Research, Bijenicka 54, Zagreb, Croatia
28. Department of Aquatic Sciences and Assessment, Swedish University of Agricultural Sciences (SLU), Box 7050, SE-750 07 Uppsala, Sweden
29. Institute of Forensic Research, Westerplatte 9, 31-033 Krakow, Poland
30. University of Lisbon, Faculty of Pharmacy, Av. Professor Gama Pinto, 1649-003 Lisbon, Portugal
31. Water and Soil Quality Research Group, Department of Environmental Chemistry, Institute of Environmental Assessment and Water Research (IDAEA-CSIC), Jordi Girona, 18-26, 08034 Barcelona, Spain
32. Department of Pharmacology and Toxicology, University of Iceland, Hofsvallagata 53, Reykjavík, Iceland
33. Institute of Legal Medicine and Core Facility Metabolomics, Medical University of Innsbruck, Innrain 52, 6020 Innsbruck, Austria
34. Queensland Alliance for Environmental Health Sciences (QAEHS), The University of Queensland, 20 Cornwall Street, Woolloongabba, Queensland, 4102, Australia
35. University of Bath, Department of Chemical Engineering, Claverton Down, Bath BA2 7AY, UK
36. Process and Systems Engineering Center (PROSYS), Department of Chemical and Biochemical Engineering, Technical University of Denmark, Building 229, 2800 Kgs. Lyngby, Denmark
37. University of Novi Sad, Faculty of Technical Sciences, Department of Environmental Engineering and Occupational Safety, Trg Dositeja Obradovica 6, Novi Sad 106314, Serbia
38. AGH University of Science and Technology, Department of Coal Chemistry and Environmental Sciences, Al. Mickiewicza 30, Krakow, Poland
39. Department of Chemical Engineering, McGill University, 3610 University, Montreal, Quebec, H3A 0C5, Canada
40. Istituto di Ricerche Farmacologiche Mario Negri IRCCS, Via Giuseppe La Masa, 19, 20156 Milan, Italy
41. Swiss Federal Institute of Aquatic Science and Technology, Eawag, Urban Water Management, Überlandstrasse 133, 8600 Dübendorf, Switzerland

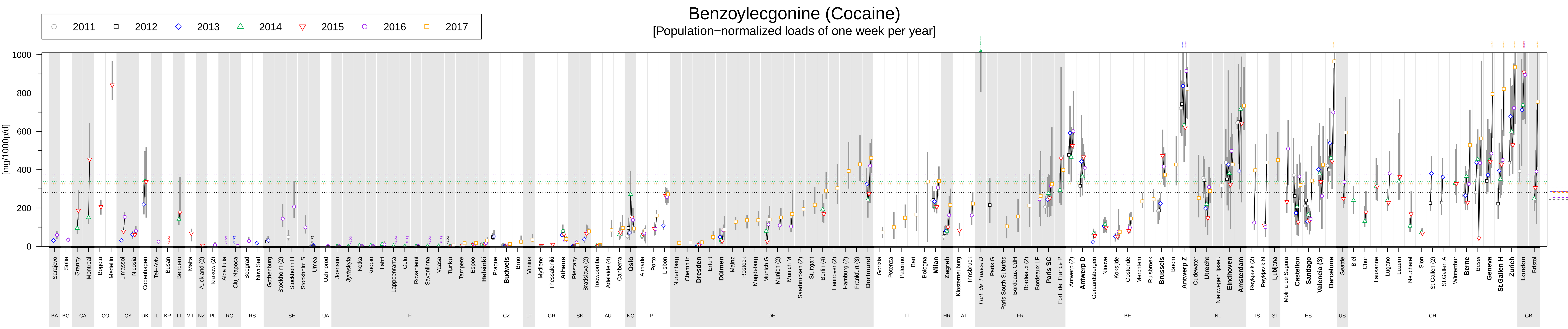

Dots: weekly mean loads. **Grey bars:** min to max range of monitored week. **Black lines:** visual aid linking data of subsequent years.

Fill color of dots: Values <LOQ (limit of quantification) were replaced with 0.5xLOQ if at least one value of the week was >LOQ; white = no values <LOQ, light grey = 1−3 values <LOQ, dark grey = 4−6 values >LOQ.

Calculation of population−weighted overall means: cities exhibiting abnormal values for at least one year (italic font, see text for details) and cities reporting '<LOQ' (for all days in one week) were excluded.

Dotted lines: overall mean loads for cities that participated five years or more (cities in bold font). **Dashed lines (right margin):** overall mean loads for all cities that participated in a particular year.

Numbers in brackets: number of wastewater treatment plants monitored in the same city (weekly mean load is the population−weighted average of all wastewater treatment plants monitored in that city).

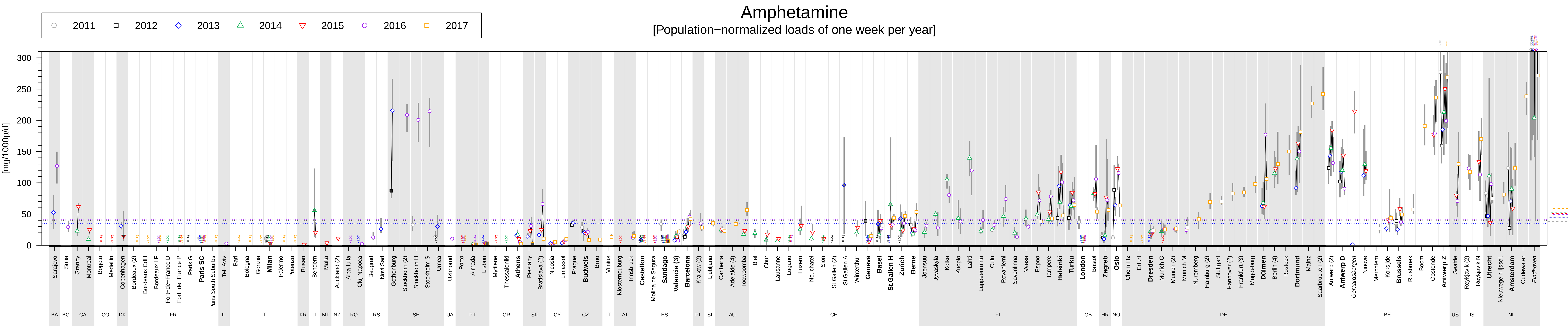

Dots: weekly mean loads. **Grey bars:** min to max range of monitored week. **Black lines:** visual aid linking data of subsequent years.

Fill color of dots: Values <LOQ (limit of quantification) were replaced with 0.5xLOQ if at least one value of the week was >LOQ; white = no values <LOQ, light grey = 1−3 values <LOQ, dark grey = 4−6 values >LOQ.

Calculation of population−weighted overall means: cities exhibiting abnormal values for at least one year (italic font, see text for details) and cities reporting '<LOQ' (for all days in one week) were excluded.

Dotted lines: overall mean loads for cities that participated five years or more (cities in bold font). **Dashed lines (right margin):** overall mean loads for all cities that participated in a particular year.

Numbers in brackets: number of wastewater treatment plants monitored in the same city (weekly mean load is the population−weighted average of all wastewater treatment plants monitored in that city).

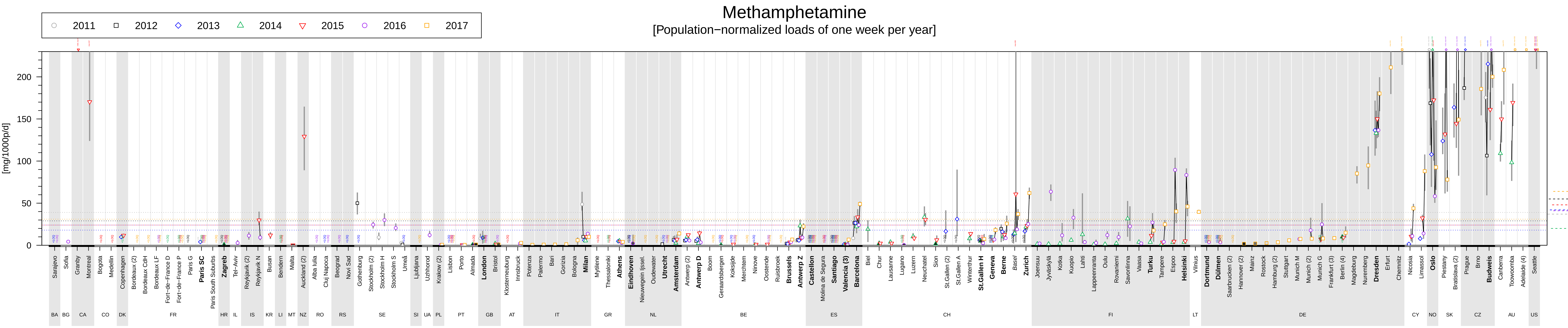

Dots: weekly mean loads. **Grey bars:** min to max range of monitored week. **Black lines:** visual aid linking data of subsequent years.

Fill color of dots: Values <LOQ (limit of quantification) were replaced with 0.5xLOQ if at least one value of the week was >LOQ; white = no values <LOQ, light grey = 1−3 values <LOQ, dark grey = 4−6 values >LOQ.

Calculation of population−weighted overall means: cities exhibiting abnormal values for at least one year (italic font, see text for details) and cities reporting '<LOQ' (for all days in one week) were excluded.

Dotted lines: overall mean loads for cities that participated five years or more (cities in bold font). **Dashed lines (right margin):** overall mean loads for all cities that participated in a particular year.

Numbers in brackets: number of wastewater treatment plants monitored in the same city (weekly mean load is the population−weighted average of all wastewater treatment plants monitored in that city).

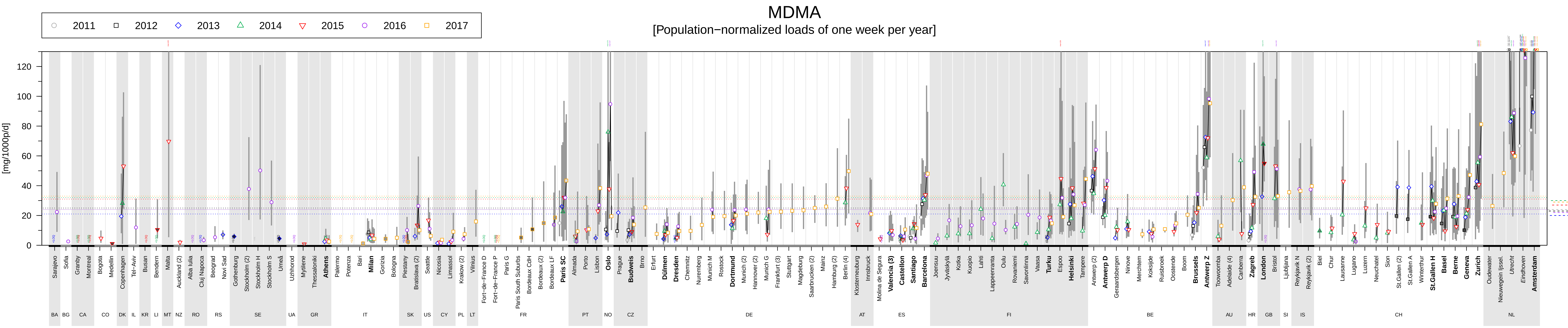

Dots: weekly mean loads. **Grey bars:** min to max range of monitored week. **Black lines:** visual aid linking data of subsequent years.

Fill color of dots: Values <LOQ (limit of quantification) were replaced with 0.5xLOQ if at least one value of the week was >LOQ; white = no values <LOQ, light grey = 1−3 values <LOQ, dark grey = 4−6 values >LOQ.

Calculation of population−weighted overall means: cities exhibiting abnormal values for at least one year (italic font, see text for details) and cities reporting '<LOQ' (for all days in one week) were excluded.

Dotted lines: overall mean loads for cities that participated five years or more (cities in bold font). **Dashed lines (right margin):** overall mean loads for all cities that participated in a particular year.

Numbers in brackets: number of wastewater treatment plants monitored in the same city (weekly mean load is the population−weighted average of all wastewater treatment plants monitored in that city).

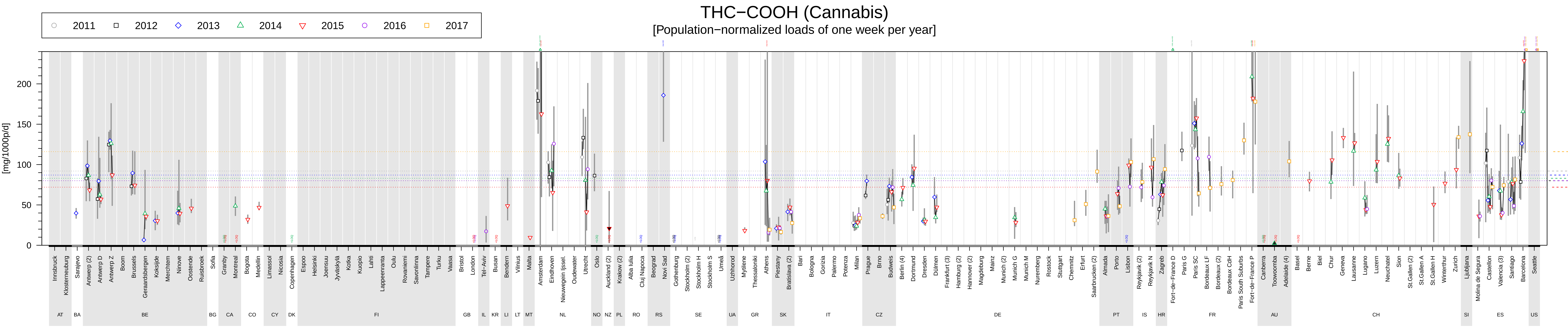

Dots: weekly mean loads. **Grey bars:** min to max range of monitored week. **Black lines:** visual aid linking data of subsequent years.

Fill color of dots: Values <LOQ (limit of quantification) were replaced with 0.5xLOQ if at least one value of the week was >LOQ; white = no values <LOQ, light grey = 1−3 values <LOQ, dark grey = 4−6 values >LOQ.

Calculation of population−weighted overall means: cities exhibiting abnormal values for at least one year (italic font, see text for details) and cities reporting '<LOQ' (for all days in one week) were excluded.

Dotted lines: overall mean loads for cities that participated five years or more (cities in bold font). **Dashed lines (right margin):** overall mean loads for all cities that participated in a particular year.

Numbers in brackets: number of wastewater treatment plants monitored in the same city (weekly mean load is the population−weighted average of all wastewater treatment plants monitored in that city).

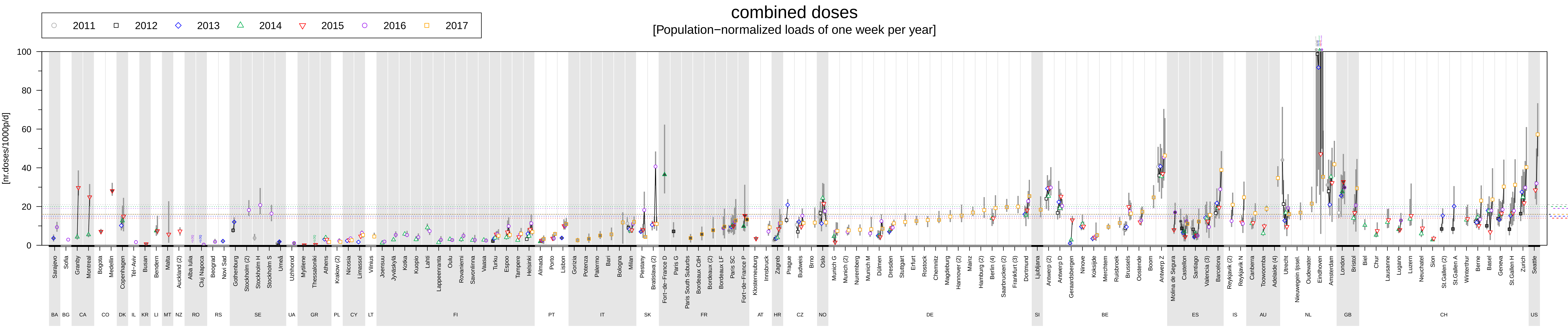

Dots: weekly mean loads. **Grey bars:** min to max range of monitored week. **Black lines:** visual aid linking data of subsequent years.

Fill color of dots: Values <LOQ (limit of quantification) were replaced with 0.5xLOQ if at least one value of the week was >LOQ; white = no values <LOQ, light grey = 1−3 values <LOQ, dark grey = 4−6 values >LOQ.

Calculation of population−weighted overall means: cities exhibiting abnormal values for at least one year (italic font, see text for details) and cities reporting '<LOQ' (for all days in one week) were excluded.

Dotted lines: overall mean loads for cities that participated five years or more (cities in bold font). **Dashed lines (right margin):** overall mean loads for all cities that participated in a particular year.

Numbers in brackets: number of wastewater treatment plants monitored in the same city (weekly mean load is the population−weighted average of all wastewater treatment plants monitored in that city).
